# The Mouse Model of Pancreatic Cancer Atlas (MMPCA) for classification of pancreatic cancer lesions: A large histological investigation of the Ptf1a^Cre/+^;LSL-Kras^G12D/+^ transgenic mouse model of pancreatic cancer

**DOI:** 10.1371/journal.pone.0187552

**Published:** 2017-11-09

**Authors:** Michelle J. Veite-Schmahl, Adam C. Rivers, Daniel P. Regan, Michael A. Kennedy

**Affiliations:** Department of Chemistry & Biochemistry, Miami University, Oxford, Ohio, United States of America; Indiana University School of Medicine, UNITED STATES

## Abstract

Pancreatic ductal adenocarcinoma (PDAC) is one of the leading forms of cancer related deaths in the United States. With limited treatment options and unreliable diagnostic methods, long-term survival rates following a diagnosis of pancreatic cancer remain poor. Pancreatic intraepithelial neoplasia (PanIN) are precancerous lesions that precede progression towards PDAC. PanIN occur in increasing complexity as the disease progresses and the description of PanIN plays a critical role in describing, staging and diagnosing PDAC. Inconsistencies in PanIN classifications exist even amongst leading pathologists. This has led to debate and confusion among researchers and pathologists involved in pancreatic cancer research, diagnosis and treatment. We have sought to initiate a discussion with leading pathologists with a goal of increasing consensus in the interpretation of PanIN and associated structures within the precancerous pancreas. Toward achieving this goal, we are in the process of conducting an extensive study of over 1000 male and female pancreata in varying stages of PanIN progression isolated from the Ptf1a^Cre/+^;LSL-Kras^G12D/+^ transgenic mouse model of pancreatic cancer. Using this extensive database, we have established the Mouse Model of Pancreatic Cancer Atlas (MMPCA) to serve as a platform for meaningful and interactive discussion among researchers and pathologists who study pancreatic disease. We hope that the MMPCA will be an effective tool for promoting a more consistent and accurate consensus of PanIN classifications in the future.

## Introduction

Pancreatic cancer is one of the most lethal cancers and is currently estimated as the third leading cause of cancer related deaths in the United States [1–4]. Pancreatic ductal adenocarcinoma (PDAC) accounts for more than 95% of all pancreatic malignancies and the 5-year survival rate of PDAC, which has historically been at ~5% [2, 5, 6], has recently improved to 9% based on the latest estimate from the American Cancer Society (https://www.cancer.org/content/dam/cancer-org/research/cancer-facts-and-statistics/annual-cancer-facts-and-figures/2017/cancer-facts-and-figures-2017.pdf). The high mortality rate of PDAC is due in part due to the typical inability to detect the disease before reaches an advanced stage. A major challenge to early detection is that the size of key precancerous lesions, known as pancreatic intraepithelial neoplasia (PanIN), are below the detection limit of current imaging capabilities [7, 8], and partially due to the cancer’s ability to resist all conventional forms of chemotherapy and radiotherapy treatment [9].

The description of PanIN plays a central role in the diagnosis and staging of pancreatic disease. PanIN have historically been described assuming a model that PanIN emerge from metaplasia of pancreatic ductal epithelia. PanIN are currently classified into three main groups, PanIN-1, PanIN-2 and PanIN-3, with PanIN-1 being further broken down into PanIN-1A and PanIN-1B. However, often a histological feature in a diseased pancreas does not fit neatly into one of the three PanIN classes, and this is where consensus begins to break down. Although the existing PanIN classification schemes are descriptive, they may not accurately reflect PanIN etiology since an alternative model for PanIN emergence from acinar-to-ductal metaplasia (ADM) is gaining acceptance [2, 10, 11]. As seen in many pancreatic cancer animal models, acinar cells lose their zymogen granules and the acinar tissue undergoes a transformation into structures that take on the appearance of duct-like structures [10, 12]. The apparent epithelial cells lining the pseudo-like lumens of these duct-like structures are frequently classified using the PanIN classification schemes. Despite the emergence of the ADM model, it is likely that not all PanIN are derived from this process [6]. Therefore, before discussing the history of the development of PanIN classification schemes, it is important to acknowledge that the current research indicates that PanIN may emerge from two distinct processes, one being origination from pancreatic ductal epithelia, and a second having origin from ADM. Although the investigation of ADM as an origin of PanIN in humans needs more examination, it appears that PanIN classification and grading should be made in the context of ADM.

In 1999, the Pancreatic Cancer Think Tank, sponsored by the National Cancer Institute, was formed and established the nomenclature and classification scheme for PanIN. Members were composed of pathologists from the United States and Europe [13]. The group was able to generate a classification scheme for various stages of PanIN, along with descriptive guidelines for identification of the different stages. Even before the classification system was adopted into practice, pathologists disagreed on the classification for PanIN-2 leading to discrepancies in classification. This prevented consistent classification of the PanIN-2 stage, which led to subsequent confusion within the field. Although pathologists were using this new scheme, they were not universally consistent.

Another group of international experts was established to further discuss the classification system. This meeting was held at Johns Hopkins Hospital in 2003 [14]. At this meeting, three tasks were pursued: first, to discuss the current classification systems; second, to discuss the obscurity of the definitions; and last, to determine revisions to the existing definitions. Although the group was able to establish solutions for some of the ambiguities, they concluded that additional guidelines and more defining features were needed to further develop a consensus of the various stages of PanIN for clinical practices and research [14]. Additionally, existing literature seems to agree with the conclusion that the stages of PanIN need to be more rigorously defined and a greater concurrence of the terminology needs to be established to reach an overall consensus [13–18]. Even though improvement has been made in this area, additional efforts are needed to improve consistency, consensus and accuracy of PanIN classifications across the field [15].

Normal ductal epithelium cells are cuboidal in appearance with no signs of mucinous cytoplasm or nuclear crowding. Squamous (transitional) metaplasia refers to transitioning of normal cuboidal epithelial cells into another distinct type of cells, as occurs during elongation of the cuboidal cells seen in PanIN-1A. PanIN-1A is distinguishable by flat, basally located nuclei within columnar cells and with mucin present. PanIN-1B is similar to PanIN-1A, but display a papillary or basally located pseudostratified structure. PanIN-2 may be present as flat or papillary and display abnormal nuclear characteristics without cribriforming luminal necrosis. PanIN-3 are typically papillary with cribriforming of the lumen and loss of nuclear polarity [13–15]. Table 1, established by the Pancreatic Cancer Think Tank, shows a more detailed outline of the classifications of the three stages [13, 14]. Although these PanIN classifications have been established, further classification and discussion is still needed.

**Table 1:** Classification System Established by the Pancreatic Cancer Think Tank.

Classification System Established by the Pancreatic Cancer Think Tank [13, 14]	
**Normal**	• Cuboidal to low-columnar epithelium with amphophilic cytoplasm • Mucinous cytoplasm, nuclear crowding, and atypia are not seen
**Squamous (Transitional) Metaplasia**	• Normal cuboidal ductal epithelium is replaced by mature stratified squamous or pseudostratified transitional epithelium without atypia
**PanIN-1A**	• Flat epithelial lesions composed of tall columnar cells with basally located nuclei and abundant supranuclear mucin • Nuclei are small and round-to-oval in shape • When oval, nuclei are oriented perpendicular to the basement membrane • It is recognized that there may be considerable overlap among nonneoplastic, flat, hyperplastic lesions, and flat, neoplastic lesions without atypia. Some may choose to designate these lesions with the modifier term “lesion” (“PanIN/L 1A”) to acknowledge that the neoplastic nature of many cases of PanIN 1A has not been established.
**PanIN-1B**	• Papillary, micropapillary, or basally pseudostratified architecture, otherwise are identical to PanIN 1A
**PanIN-2**	• Mucinous epithelial may be flat, but mostly papillary • Must have some nuclear abnormalities, which can include: some loss of polarity, nuclear crowding, enlarged nuclei, pseudostratification, and hyperchromatism • Mitoses are rare, but when present are nonluminal (not apical), and are not atypical • True cribriform structures with luminal necrosis and marked cytologic abnormalities are not seen, and when present, should suggest the diagnosis of PanIN 3
**PanIN-3**	• Usually papillary or micropapillary, may rarely be flat • Ture cribriforming, the appearance of “budding off” of small clusters of epithelial cells into the lumen, and luminal necrosis • Cytologically, these lesions are characterized by a loss of polarity, dystrophic goblet cells (goblet cells with nuclei oriented toward the lumen and mucinous cytoplasm oriented toward the basement membrane), mitoses that may be abnormal, nuclear irregularities, and prominent (macro) nucleoli • Resemble carcinoma at the cytonuclear level, but invasion through the basement membrane is absent

An alternative classification system has been introduced [18] based on a meeting held at Johns Hopkins University School of Medicine in 2014. Their aim was to discuss the current classification system and present an alternative system for easier transparency between research studies and clinical practices. Members in attendance consisted of international experts within the field. The resulting system was a two-tiered classification system, low vs. high grade. The rationale for this system was based off the absence of reporting low grade PanIN lesions in pathology reports due to their high frequency, whereas, high grade PanIN are reported because of their status as potential markers for invasive carcinoma. From the first established system, PanIN-1 & PanIN-2 would be classified as low grade PanIN, and PanIN-3 would be classified as high grade in the new two-tiered system.

Here, we have used a mouse model to investigate progression of pancreatic cancer and PanIN initiation. Mouse models of pancreatic cancer have been developed that appear to recapitulate all recognized features observed in human pancreatic cancer progression, from the precancerous PanIN precursor state to invasive carcinoma [6]. The most commonly used mouse models rely on activation of an oncogenic Kras mutant, specifically G12D Kras, that is silenced in a lox-stop-lox cassette. The oncogenic K-ras is unsilenced by placing a Cre recombinase gene under the control of a pancreas-specific promoter, namely, either the Pdx1 or Ptf1a. When expression of genes under the control of Pdx1 or Ptf1a promoters are initiated, this causes expression of the Cre recombinase specifically in the pancreas. This leads to unsilencing and subsequent expression of the oncogenic G12D Kras gene. The two models differ in the time of expression of the Pdx1 and Ptf1a promoters. At day 8 in the fetal mouse, the first pancreatic progenitor cells are identifiable. At embryonic day 8.5 in the fetal mouse, Pdx-1 expression is initiated, whereas Ptf1a expression occurs a full day later at embryonic day 9.5, committing cells to a pancreatic fate [6]. As a consequence of the differential time of initiation of Pdx1 and Ptf1a expression, we and others have found that activation of the G12D Kras under control of the Pdx1 promoter can lead to non-pancreas-specific tumors because some pancreas progenitor cells in which Pdx1 is activated on embryonic day 8.5 do not have a strict fate of belonging to the pancreas tissue. Specifically, Hingorani et al. have reported observation of mucocutaneous papillomas, intestinal metaplasia of the gastric epithelium and hyperplastic polyps of the duodenum using the PDX-1^Cre/+^ model [6], and we also observed ocular and anal mucocutaneous tumors. In contrast, Hingorani et al. reported [6], and we also observed, that activation of the G12D Kras a full day later at embryonic day 9.5 under control of the Ptf1a promoter leads to tumors exclusively restricted to the pancreas. Therefore, to ensure that tumor progression originated exclusively in pancreas tissue, the mice investigated in this study involved activation of the G12D Kras oncogene indirectly under the control of the Ptf1a promoter, and therefore the Ptf1a^Cre/+^;LSL-Kras^G12D/+^ mouse strain was used as a model of human pancreatic cancer. It should also be noted that in the adult mouse, the islet cells contain Pdx-1 expression, while the acinar cells contain Ptf1a expression [6].

Regarding the relevance of the Ptf1a^Cre/+^;LSL-Kras^G12D/+^ mouse strain as a model for human pancreatic cancer development, it should be noted that Kras mutations have been reported to be present in >90% of human pancreatic adenocarcinomas and found in more than 95% of the earliest pre-cancerous PanIN-1 features [8]. In that same study, it was reported that while the G12D Kras mutation is by far the most prevalent Kras mutation in human PanIN, G13 and Q61 mutations were also occasionally observed but at much lower frequencies [8]. Given these statistics, it appears that activation of the Kras oncogene appears to be a critical, if not universal, mutation in human pancreatic cancer. Notwithstanding, activating mutations of the Kras gene often occur in conjunction with mutations that inactivate tumor suppressor genes leading to tumor progression in many human cancers, and in most, if not all, human pancreatic cancers, mutations in multiple and different genes are often observed [19]. Consequently, the complexity and diversity of human pancreatic cancers will naturally not be fully represented in this mouse model of pancreatic cancer. However, the Ptf1a^Cre/+^;LSL-Kras^G12D/+^ strain used in this study has been shown by others, and observed in our lab, to progress from a PanIN pre-cancerous stage all the way through tumor formation, which seems to closely mimic pancreatic cancer progression in humans [6,20–22].

The goal in creating the Mouse Model of Pancreatic Cancer Atlas (MMPCA) is to utilize our extensive histology database to establish an extensive resource in which researchers and pathologists in the field of pancreatic cancer can engage in interactive conversations about the PanIN classification and broader interpretation of pancreas tissue features involved in pancreas pathology. Images for the 1^st^ release of the MMPCA were obtained from over 500 male and female mice at ages ranging from 5- to 16-months from the Ptf1a^Cre/+^;LSL-Kras^G12D/+^ mouse model. The MMPCA provides multiple images from different mice at multiple magnifications and in the form of an open and continuous discussion. By providing an online hub with one of the most extensive publicly accessible and annotatable histology databases available for a mouse model of pancreatic cancer, experts will be able to discuss their opinions on classification patterns and interactively address classification discrepancies in specific histology images. After this interaction takes hold, the online atlas will allow for researchers in the field to collaborate on refining future guidelines for classification of PanIN and other features observed in pancreatic cancer tissues. The MMPCA will also enable comparison with what is known about pancreatic cancer progression in humans, and as more data becomes available, allow further assessment of the Ptf1a^Cre/+^;LSL-Kras^G12D/+^ mouse strain as a model for human pancreatic cancer.

## Materials and methods

### Mouse handling and euthanasia

The Institutional Animal Care and Use Committee (IACUC) at Miami University approved the procedures performed in this study as documented by IACUC project numbers 854 and 855. The protocols were approved by both the ethics committee and the IACUC of Miami University (Animal Welfare Assurance Number: D16-00100). All surgeries were performed under isoflurane anesthesia, and all efforts were made to minimize animal suffering. All authors and personnel involved were required to complete online and in class animal training prior to any work involving animals. All dissections were conducted as previously described [23]. The procedure began by placing the mouse into the anesthesia chamber, a glass jar with an isoflurane soaked pad and lid, for approximately one minute until the mouse was unconscious. A foot pinch stimulus test was conducted to ensure discomfort was not being experienced. If discomfort was displayed, the mouse was placed back into the glass jar until the stimulus test was passed. Cervical dislocation or removal of the heart was then conducted. Mice that displayed abnormal behavior prior to sacrifice, such as: inactivity, refusing to eat and/or drink, or discomfort, were said to have reached an experimental endpoint and were immediately removed through approved protocol procedures. Mice were allowed to reach pre-determined age categories prior to sacrifice, unless the endpoint listed above was reached prior. For this study, a total of 30 mice were used, 9 study mice and 1 control mouse for each age category (5, 11, and 15), all reaching their designed sacrifice date. At the time of sacrifice, all animal welfare considerations were taken, including efforts to minimize distress and suffering.

### Mouse model and genotyping

The Ptf1a^Cre/+^;LSL-Kras^G12D/+^ mouse model of pancreatic cancer was used in this study. Control mice were represented by Ptf1a^Cre/-^;LSL-Kras^G12D/-^ mice and study mice were represented by Ptf1a^Cre/+^;LSL-Kras^G12D/+^ mice. Mice were acquired from The Jackson Laboratory and then bred in-house at Miami University to obtain the desired number needed for the study. The KrasG12D mice, scientific strain name B6.129-Kras<tm4Tyj>, contains a mutant Kras that is silenced by a Lox-stop-Lox cassette. The mutation is unsilenced through the crossbreeding with Ptf1a mice, specific strain name B6.Ptf1a(tm1.1(cre)Cvw), that express cre-recombinase under the control of the Cre knockin at the Ptf1a-p48 locus, which is primarily expressed in the pancreas. Genotyping for the Kras mutation and Cre-recombinase was conducted to determine the classification of each mouse. DNA from each mouse was obtained through an ear punch for later extraction and also for identification purposes throughout the study.

DNA was extracted through the use of a thermocycler, Gene Amp PCR System 9700, after submerging the ear clipping in 25 uL of 25 mM NaOH/0.2 mM EDTA at a pH of 12. After an hour at 98°C, the sample was neutralized with 25 uL of 40 mM Tris-HCl at a pH of 5. The sample is stored at -20°C until PCR amplification of the Kras, Cre, and control primers are conducted. The extracted DNA was added to a mixture containing Dreamtaq Green Master Mix (Fisher Scientific), MgCl_2_ (Fisher Scientific), water, and either the Kras, Cre, or control primers (IDT). For the Kras program, the mixture was denatured at 94°C for 30 seconds, then annealed at 69°C for 1 minute, and elongated at 72°C for 1 minute using the Gene Amp PCR System 9700 thermocycler for 35 cycles, then held at 4°C when complete. For the Cre program, the mixture was denatured at 94°C for 1 minute, then annealed at 63°C for 20 seconds, and elongated at 72°C for 45 seconds using the thermocycler for 30 cycles, then held at 4°C when complete.

An agarose gel stained with ethidium bromide was used to separate the amplified gene product through gel electrophoresis. A UV light on the UVP ChemiDoc-It 2 815 Imager was used to show the presence or absence of the amplified DNA. When present, the Kras mutation is shown at 550 bp and the Cre at 250 bp. Classification as a study mouse means both the Kras and Cre were present, and classification as a control mouse means both the Kras and Cre were absent. In this atlas, cohorts of nine study mice and one control mouse per age category were generated for age groups 5-, 11-, and 15- months. The Institutional Animal Care and Use Committee (IACUC) at Miami University approved all procedures conducted in this research.

### Study size

Collectively, over 1000 study (Ptf1a^Cre/+^;LSL-Kras^G12D/+^) and control (Ptf1a^Cre/-^;LSL-Kras^G12D/-^) mice were used in this study. Starting at age 5-months, 24 mice of each gender for both control and study mice were sacrificed and pancreata harvested for histological analysis extending out to 15-months of age. The complete distribution of mice utilized in the study, and therefore representing the MMPC Atlas, is summarized in Table 2.

**Table 2:** Distribution of Gender, Age, and Numbers of Mice used in the MMPCA.

Age	Control		Study	
	Male	Female	Male	Female
5-months	24	24	24	24
6-months	24	24	24	24
7-months	24	24	24	24
8-months	24	24	24	24
9-months	24	24	24	24
10-months	24	24	24	24
11-months	24	24	24	24
12-months	24	24	24	24
13-months	24	24	24	24
14-months	24	24	24	24
15-months	24	24	24	24
Total	264	264	264	264

### Pancreatic histology

Initial entries in the Mouse Model of Pancreatic Cancer Atlas (MMPCA) were generated from groups of 5-, 11-, and 15-month old mice. Histology images from these mice were used to determine PanIN grades and frequencies in the study mice in comparison to normal pancreatic tissue. It has been previously reported that more than 80% of the ducts are expected to be normal at approximately 2.25 months, 68% of the ducts are expected to be normal from age 4–5 months, and 18% of the ducts are expected to be normal for mice in the age range from 7–10 months [6]. Mice were dissected following the procedures previously described [23]. Briefly, mice were euthanized once the targeted age was reached, and pancreata were collected from each mouse by dissection and placed into formalin overnight at room temperature. The fixing solution for the pancreata was then changed to 70% ethanol and stored at 4°C until processing. A Leica TP 1020 was used to process pancreatic tissue, and the Shandon Histocentre 3 was used to embed the pancreatic tissue. A Thermo-Shandon Finesse ME Microtome was used to produce 5 μm sections that were placed on slides for staining.

Slides were stained with hematoxylin and eosin. First, the slides were placed in xylene substitute for 4 minutes. Then, the slides were transferred to absolute ethanol for 4 minutes, 95% ethanol for 2 minutes, and 70% for 2 minutes. They were then rinsed with tap water for 4 minutes and kept between 37–40°C to help distinguish the blues of the hematoxylin. They were placed in the hematoxylin (modified with acetic acid, mercury free) for 1 minute and were placed back into the tap water for 4 minutes. Then the slides were dipped for a second in the 1% acid alcohol solution and were rinsed again in the tap water for 4 minutes. After being placed for 2 minutes in “Scott’s Tap” water, a combination of distilled water, sodium bicarbonate, and magnesium sulfate, they were rinsed in tap water for 4 minutes. They were then placed in eosin for 30 seconds and put into 95% ethanol for 6 minutes. The slides were then placed into absolute ethanol for 6 minutes, then xylene substitute for 4 minutes. The undersides of the slides were wiped free from solution and 2 drops of permount was added with a coverslip. Images were then taken through the use of an Olympus AX70 Light Microscope in the Center for Advanced Microscopy and Imaging at Miami University.

### Online atlas

**Fig 1:**
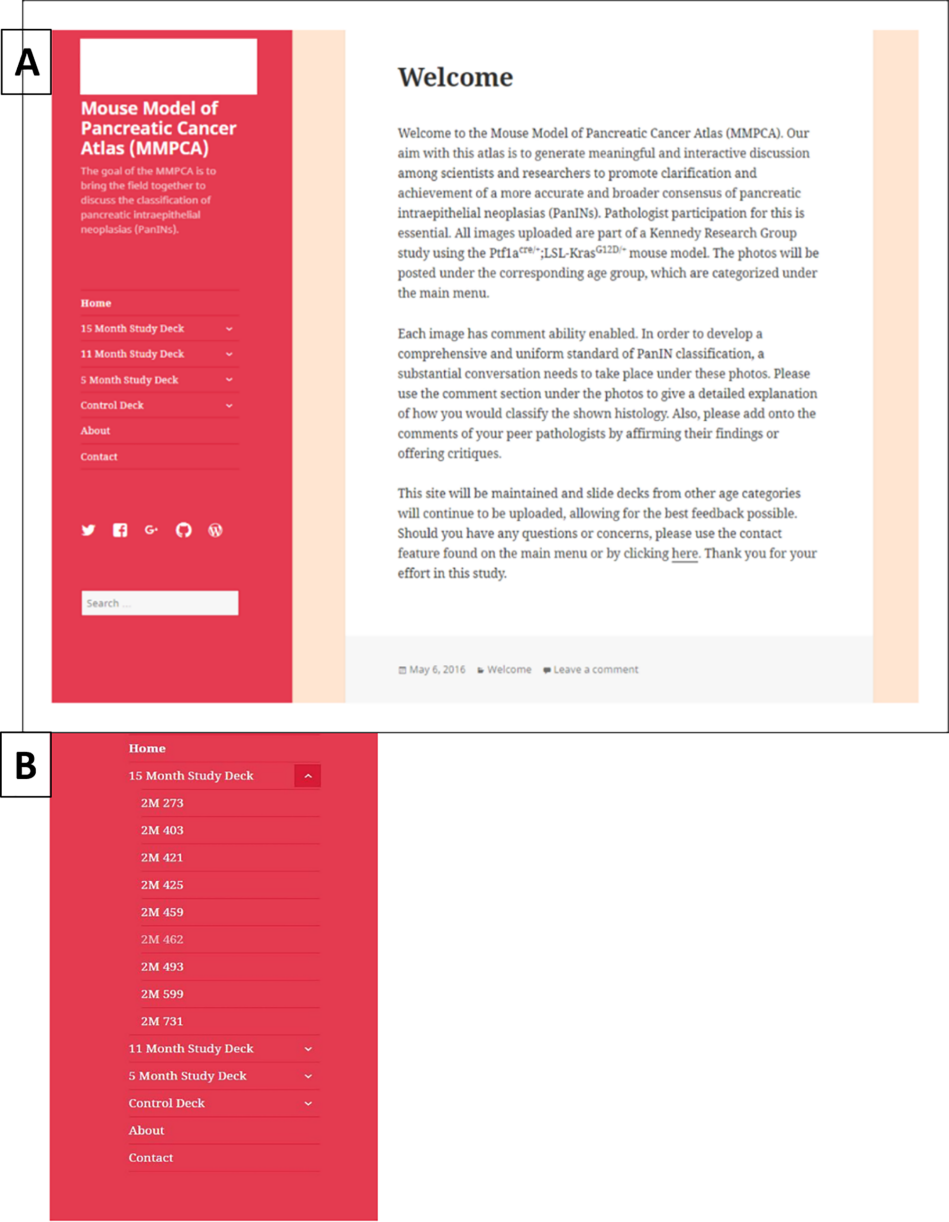
Mouse Model of Pancreatic Cancer Atlas (MMPCA). (A) Homepage of the MMPCA on WordPress that shows welcome information, slide deck categories, an about page, and contact. The comment ability is shown at the bottom of the image titled, “Leave a comment.” (B) Content of the atlas slide decks with 15-month study deck displaying mice numbers.

**Fig 2:**
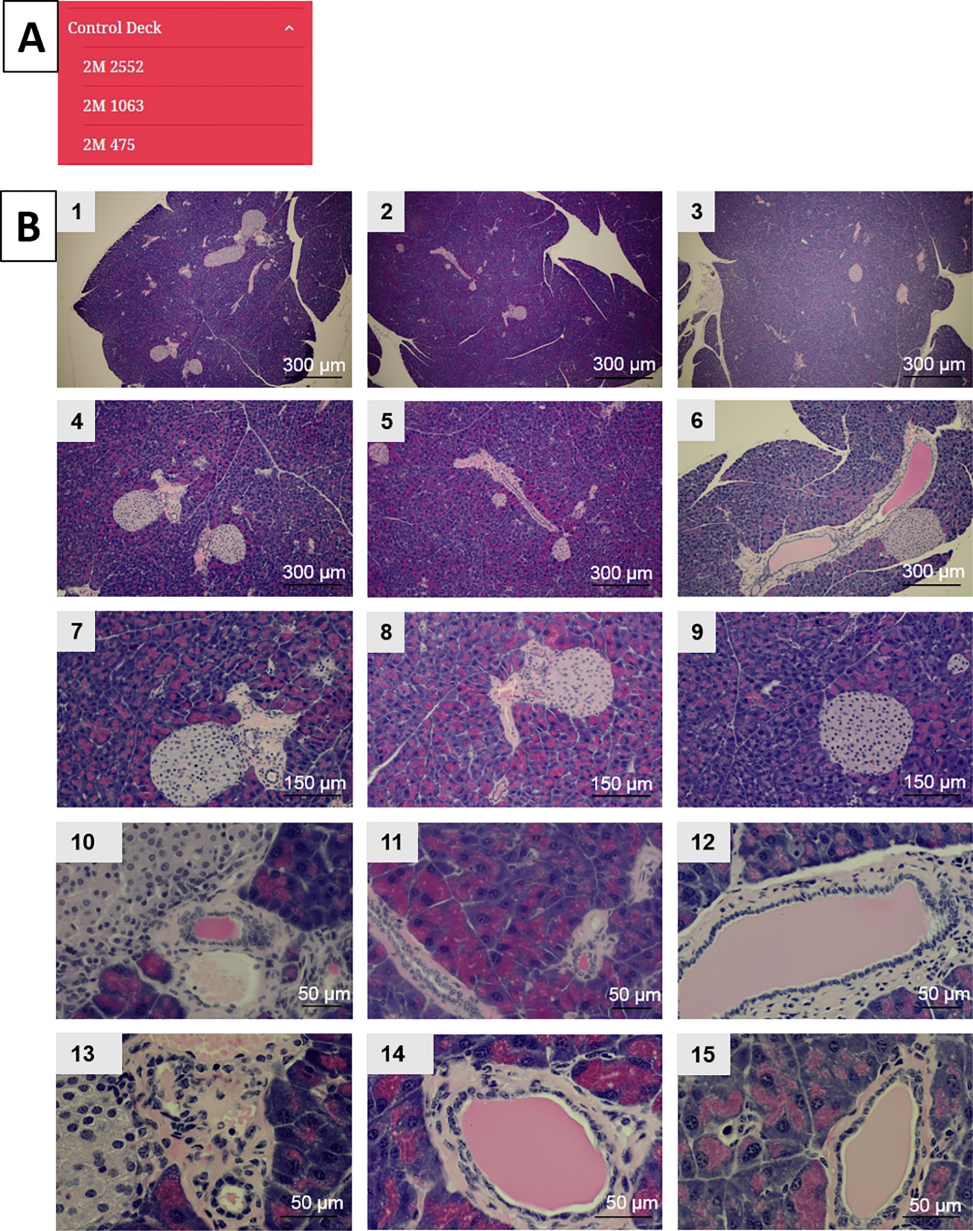
Control slide deck. (A) Content of the control mice image slide deck showing an example from each age group of 5-, 11-, and 15-months. (B) Images from the control deck displaying images at different magnifications from the same mouse at 5-months-of-age (1, 4, 7, 10, 13), 11-months-of-age (2, 5, 8, 11, 14), and 15-months-of-age (3, 6, 9, 12, 15).

**Fig 3:**
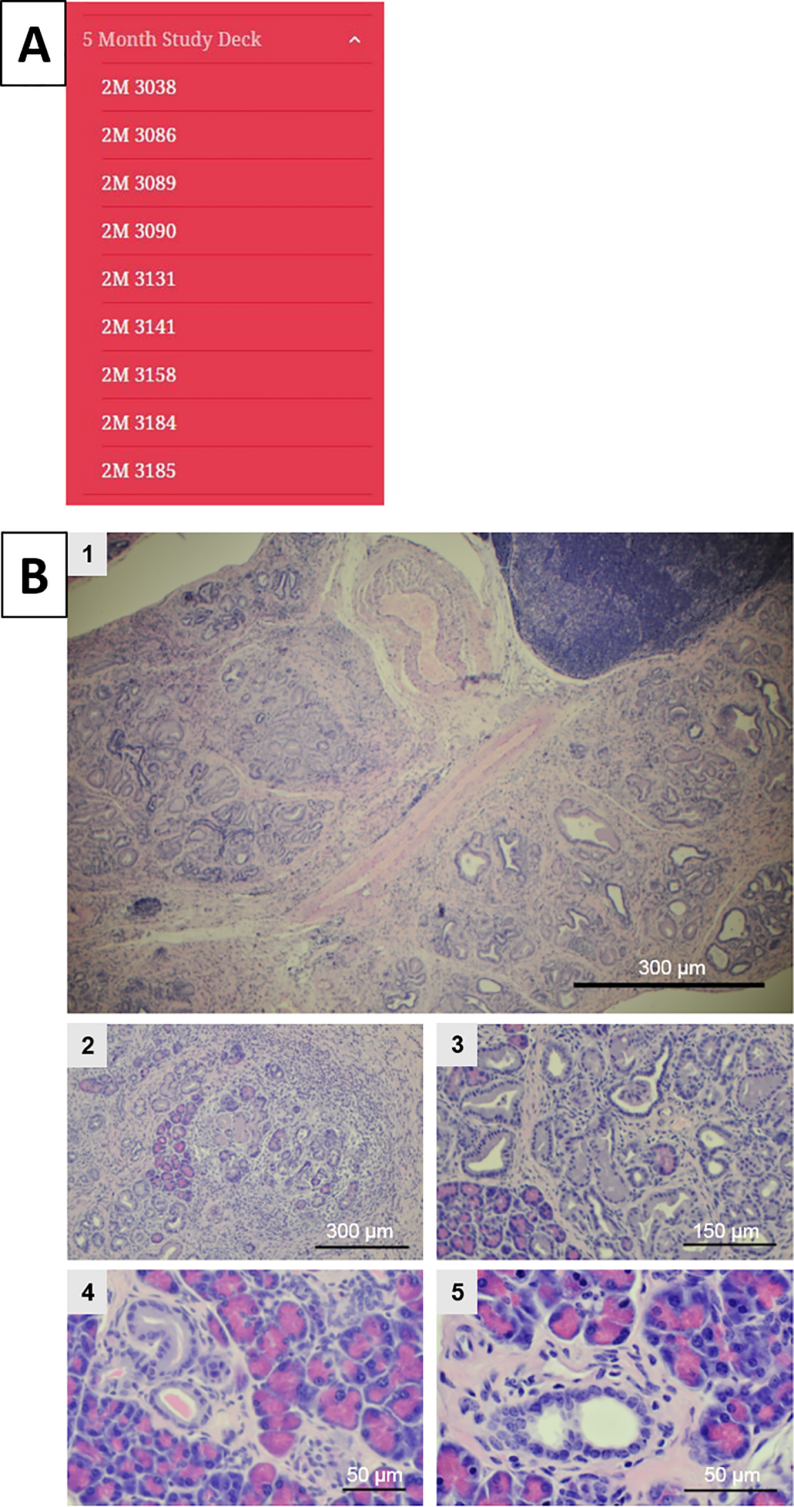
Slide deck for 5-month-old mice and comment ability. (A) Content of the 5-month study deck. (B) Example images from the atlas at 5 months displaying an example of each magnification taken (1 at 4X, 2 at 10X, 3 at 20X, 4 at 40X, and 5 at 60X).

**Fig 4:**
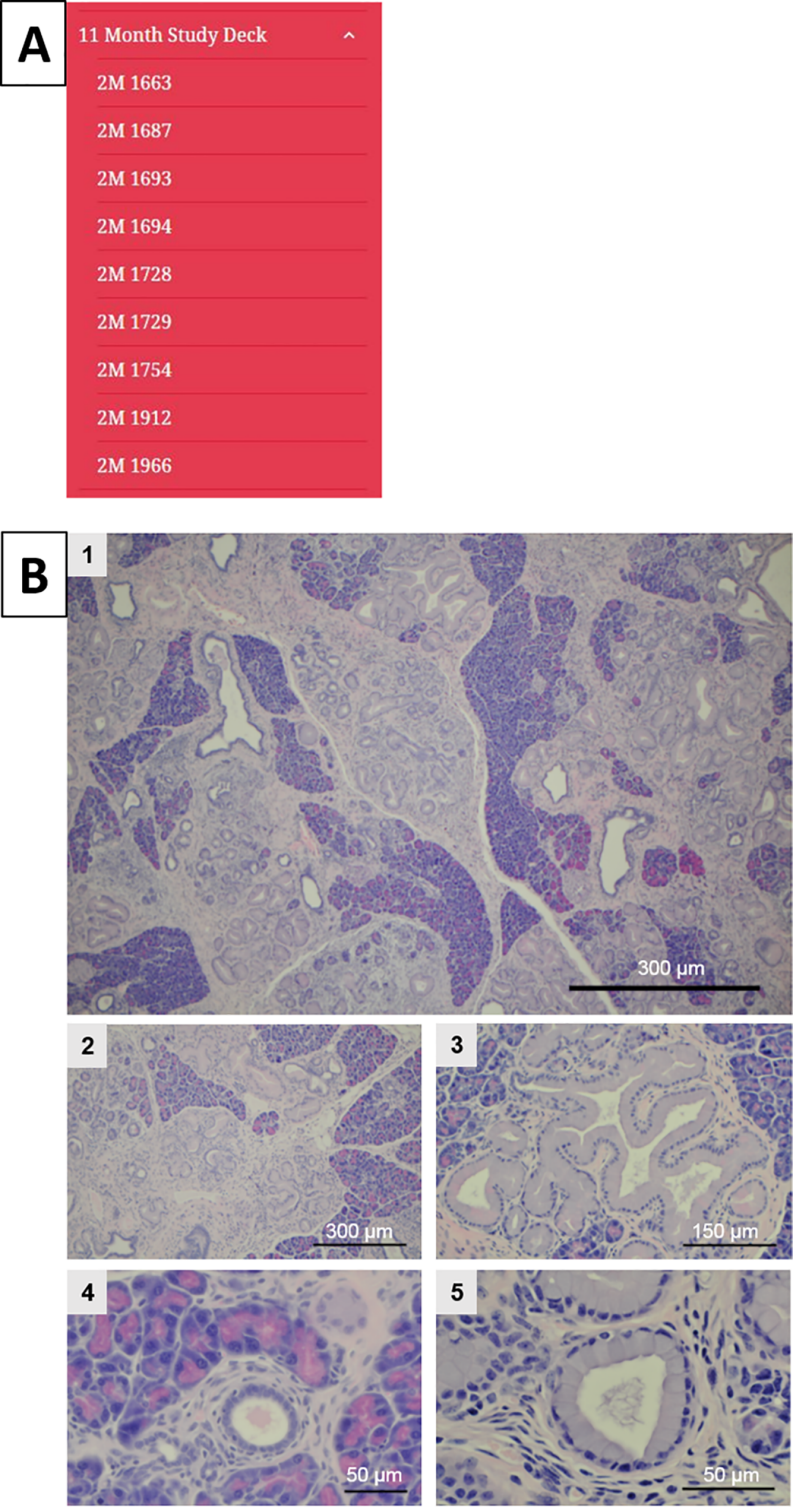
Slide deck for 11-month-old mice. (A) Content of the 11-month study deck. (B) Example images from the atlas at 11 months displaying an example of each magnification taken (1 at 4X, 2 at 10X, 3 at 20X, 4 at 40X, and 5 at 60X).

**Fig 5:**
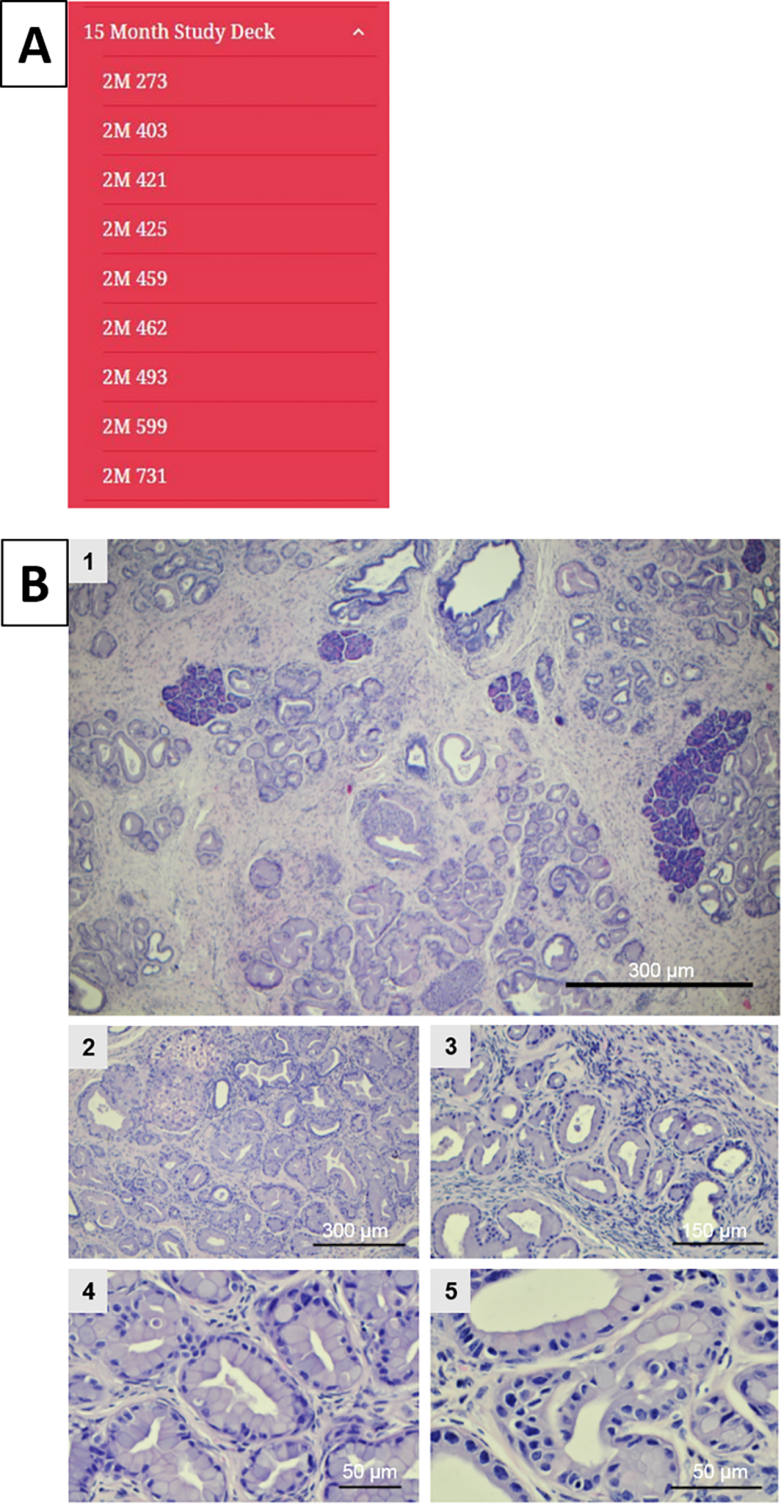
Slide deck for 15-month-old mice. (A) Content of the 15-month study deck. (B) Example images from the atlas at 15 months displaying an example of each magnification taken (1 at 4X, 2 at 10X, 3 at 20X, 4 at 40X, and 5 at 60X).

Once all of images were captured with the Olympus AX70, they were then uploaded into “slide decks” on a WordPress page established for the online atlas. The homepage for the MMPCA is shown in Fig 1A. The online atlas was created solely for the intent of establishing a hub for pathologists and collaborators to have access to the extensive histology database. The site was created so that each slide deck, i.e. a compilation of slide images corresponding to a specific age group, has its own page, example shown in Fig 1B. Slide decks are composed of microscope images for each histology section generated at multiple magnifications at 4X, 10X, 20X, 40X, and 60X lens magnifications. The initial slide decks are comprised of nine study mouse pancreata samples and one control mouse pancreata per age category. Each individual image was cataloged and labeled under the corresponding slide deck so that each study and control model had its own sub-page under the slide deck, example shown in Fig 1B. Once on the page of the study (or control) mouse model number, all histology images belonging to that model are viewable; examples of images from the atlas at varying ages are shown in Figs 2–5. This structure allows for the pathologists and collaborators to comment on individual images to discuss PanIN grading, hallmark features, or discuss any discrepancies with other experts on that particular image, example of commenting ability shown in Fig 1A. The online atlas is open-access and publically available at https://mousepancreaticcancer.wordpress.com/ and is under the control and maintenance of the Kennedy Research Group at Miami University. Currently, images from 5-, 11-, and 15-month-old mice are available on the database, and with the continued maintenance under the Kennedy group, additional age categories will be added and a new MMPCA version released each year.

## Results and discussion

The MMPCA is an online freely accessible atlas intended to facilitate broad participation of researchers and pathologists who are actively involved in pancreatic disease research in an online interactive conversation regarding classification of features that arise in the histological analysis of the pancreas in the Ptf1a^Cre/+^;LSL-Kras^G12D/+^ transgenic mouse model of pancreatic cancer. A screen-shot of the home welcome page of the MMPCA is shown in Fig 1A. The MMPCA for mouse pancreatic cancer classification was created to facilitate refinement and improvement of classification of PanIN and other tissue features observed in the disease pancreas. There are currently two well-known classification systems that are being used. Although these systems are similar, multiple classifications generate ambiguity among pathologists and their results. One classification system establishes PanIN-1 through PanIN-3 with details on the types of cells present or absent within each stage and is generally used more in research studies. The other classification system is a two-tiered system, low grade and high grade, which is typically used in clinical practices.

Currently, there are websites that offer in depth examples of the various classifications and subsequent stages, but none of these websites function as interactive databases that provide multiple images from multiple subjects at varying magnifications. By allowing pathologists to make comments on varying features within an image, discussion is generated, and thus conflict resolution is initiated. Multiple magnifications are also displayed to show varying features throughout the pancreatic tissue.

The initial MMPCA has been established using images from mice ages 5, 11, and 15 months to ensure that early, intermediate, and late PanIN stages were represented. Control pancreas tissue is present from each age category to establish a basis for comparison with normal tissue within this mouse model. By providing multiple images from different mice, the database provides a comprehensive model of PanIN development that seeks to resolve ambiguity in the field. From this, we hope to use our ample amount of histology from the ages 5, 11, and 15 months to help set a standard classification system of the PanIN to be used in both research studies and clinical practices.

## Conclusions

We introduce the MMPCA, an online and interactive resource for researchers and pathologists active in the investigation and classification of pancreatic cancer. The MMPCA is being populated from histology images prepared from pancreata collected from over 1000 age-matched and gender-matched Ptf1a^Cre^;LSL-Kras^G12D^ mice (>500 Ptf1a^Cre/+^;LSL-Kras^G12D/+^ and >500 Ptf1a^Cre/-^;LSL-Kras^G12D/-^). The MMPCA provides a large database of images at multiple magnifications, making vast amounts of information available to researchers that can be investigated and shared among experts around the world. The MMPCA will provide an opportunity for investigators and pathologists to refine current PanIN and pancreatic cancer classification schemes and the possibility for increasing the consensus regarding PanIN classifications and descriptions of pancreatic cancer pathology. The online and interactive nature of the MMPCA will make it possible for pathologists and researchers from around the world to participate in the dialogue and to contribute their expertise to the project by simply accessing the MMPCA web page, adding comments and interacting with other investigators.
